# Are Older Adults Less Embodied? A Review of Age Effects through the Lens of Embodied Cognition

**DOI:** 10.3389/fpsyg.2017.00267

**Published:** 2017-02-27

**Authors:** Matthew C. Costello, Emily K. Bloesch

**Affiliations:** ^1^Department of Psychology, University of Hartford, West HartfordCT, USA; ^2^Department of Psychology, Central Michigan University, Mount PleasantMI, USA

**Keywords:** embodiment, aging, cognition, perception, embodied cognition, older adults, gerontology

## Abstract

Embodied cognition is a theoretical framework which posits that cognitive function is intimately intertwined with the body and physical actions. Although the field of psychology is increasingly accepting embodied cognition as a viable theory, it has rarely been employed in the gerontological literature. However, embodied cognition would appear to have explanatory power for aging research given that older adults typically manifest concurrent physical and mental changes, and that research has indicated a correlative relationship between such changes. The current paper reviews age-related changes in sensory processing, mental representation, and the action-perception relationship, exploring how each can be understood through the lens of embodied cognition. Compared to younger adults, older adults exhibit across all three domains an increased tendency to favor visual processing over bodily factors, leading to the conclusion that older adults are less embodied than young adults. We explore the significance of this finding in light of existing theoretical models of aging and argue that embodied cognition can benefit gerontological research by identifying further factors that can explain the cause of age-related declines.

## Introduction

Embodied cognition theory argues that cognitive and perceptual processes are grounded in the organism’s sensorimotor capacities. It rests upon a rejection of the classic cognitivist division between mind and body, and holds that the two are intertwined in often unexpected ways (Foglia and Wilson, 2013). Traditional (non-embodied) models of cognition operate through manipulation of symbolic mental representations governed by logical and computational rules. Under this traditional model, the body is not given a privileged position; it is merely the means by which sensory-level inputs are stored and later processed as mental representations. These representations are understood conceptually as independent of the physicality of the body. Embodied cognition rejects this traditional account, and argues that the physicality of the body in action is not merely a vehicle for computational processes, but rather is a co-producer of cognitive processes.

Embodied cognition is generally viewed as a broad theoretical framework for understanding mind-body relations, rather than as a singular theory serving to generate explicit hypotheses. It does not employ any singular experimental methodology, but instead serves as a backdrop to help interpret existing research such as in cognitive, perceptual, and bodily action studies. In this regard, embodied cognition theorists generally draw upon traditional ‘non-embodied’ research, and therefore may be understood as largely consonant with traditional cognitive methodologies (Goldman, 2012)^1^. However, insofar as it rejects some of the central tenets of classical cognitivist theory, embodied cognition retains something of an outsider status in cognitive science. Indeed, for some the ambiguous nature of embodied cognition makes it too difficult to effectively employ (Aizawa, 2015; Mahon, 2015). However, embodied cognition has become increasingly accepted as a viable theoretical option for researchers investigating sensorimotor function and situated cognition (Creem-Regehr and Kunz, 2010; Glenberg et al., 2013; Barsalou, 2016).

Embodied cognition would seem to be a natural fit for gerontology, as aging typically manifests as concurrent physical and cognitive changes (Roberts and Allen, 2016). Physically, older adults suffer from a range of changes to the body and action systems. Older adults experience height decreases, postural changes, decreased bone density, and increased bone brittleness (DiPietro, 2001). The quantity and quality of the muscular system show marked declines with advanced age (Metter et al., 1999), with reductions in muscle mass, strength, and mobility (Visser et al., 2002). These bodily changes are matched by decreases in motor function (cf., Seidler et al., 2010), with older adults exhibiting slower motor responsiveness (Falkenstein et al., 2006), more variable and slower physical movements (Mau-Moeller et al., 2013), and reduced gait speed (Studenski et al., 2011). Mobility is decreased in older adults (Tinetti, 1986), and physical actions are complicated by declines in balance control (Laughton et al., 2003). Accordingly, older adults exhibit decreased walking stability (Lord and Menz, 2000; Lord and Sturnieks, 2005) and increased falls (Kannus et al., 2005).

Cognitively, even healthy older adults exhibit a number of changes, including declines in working memory (Zacks, 1989), selective attention (Verhaeghen and Cerella, 2002), and response inhibition (Kramer et al., 1994). Perhaps the most expected age-related deficit in cognitive processing is in processing speed, with widespread slowdowns in basic cognitive and motor performance (Salthouse, 1996). Cognitive slowdowns for seniors are especially evident in visuospatial processing (Jenkins et al., 2000); relatedly, older adults exhibit declines in visuospatial reasoning and spatial navigation (Moffat, 2009; Klencklen et al., 2012). Interestingly, cognitive slowdowns have been linked to the type of physical activity levels described above (Kraft, 2012), and both cardiovascular health (Hillman et al., 2008) and walking speed (Atkinson et al., 2007) are predictive of cognition in older adults.

In short, there is ample evidence that for older adults, the mind and body are interconnected in a manner that resonates with embodied cognition theory. However, gerontological research has largely avoided the direct application of this framework. The goal of this review is to introduce the embodied cognition framework to the field of gerontological research and to argue that it can be used to explain a wide variety of cognitive aging effects. Given that embodied cognition is a broad field of study, the scope of this review article will be limited to addressing how aging affects three subdomains of specific relevance to embodied cognition: sensory processing, mental imagery, and the perception-action link. By way of preview, a central conclusion from this review is a finding common across all three of these subdomains: compared to young adults, older adults exhibit (1) an increased reliance on visual processing (what we will call a visual dominance effect), and (2) a decreased reliance on bodily (kinesthetic, tactile, proprioceptive) factors. At the end of this review, we will discuss the significance of this finding and how it may affect existing models of sensorimotor processing in older adults.

## Foundations of Embodiment

Embodied cognition theorists have argued that sensorimotor factors serve as the groundwork for cognition. For instance, concepts have been characterized as grounded in situated, bodily action and are instantiated by neural reactivations of sensorimotor cortices used in active sensorimotor function (Barsalou, 2008). This embodied view of cognition is generally viewed in contradistinction to traditional cognitivist models in which cognitive processing operates through amodal and abstract mental representations that are conceptually separated from the sensory modalities (Fodor et al., 1975). Because embodied cognition theory argues that higher-level cognition is grounded in sensorimotor processing, it would predict that any changes at this earliest stage will result in corollary changes in more complex cognition. Accordingly, we start our review with age-related sensory changes.

### Sensory Perception and Integration

While the sensory system is subject to age effects across all five senses (Fozard and Gordon-Salant, 2001), our focus will be on declines in visual and tactile ability. Vision and tactition are particularly important senses for embodied cognition, as they provide information about the surrounding environment in which the body is located: vision provides accurate spatial feedback about both near- and far-body space, and tactition provides real-time feedback about the progress and success of physical interactions. Both sensory modalities exhibit decreased sensitivity with advanced age, with documented age-related declines in vision (Owsley, 2011) and tactile processing (Wickremaratchi and Llewelyn, 2006).

Although age-related unimodal sensory declines are of obvious importance, our perception of the world typically involves more than one sensory modality. Multisensory integration (MSI) is the combination of two or more sensory modalities into a unified percept, a process driven by a neuronal system designed to process multiple sensory inputs simultaneously (Calvert et al., 2004). MSI is particularly important for embodied cognition: in order to have knowledge of the body’s motor capabilities and the origin of sensory stimuli, there must be seamless integration of both external stimuli (visual and tactile information) and internal representations (motor commands, proprioceptive processing, and vestibular control). Thus, any change in the ability to integrate multiple sensory inputs carries with it changes in the accuracy of embodied representations.

Research indicates that younger and older adults both benefit from multisensory inputs (compared to unisensory inputs) when those inputs provide redundant information, yet this benefit is even greater in the older adults (cf., Freiherr et al., 2013). For instance, Laurienti et al. (2006) had participants discriminate between red and blue stimuli that were presented under both unimodal (either visually as colored circles or auditorily as the verbalized color words) and bimodal (both auditory and visual presentations) conditions. Although all participants evidenced reaction time facilitation in the bimodal compared to unimodal conditions, the gain was significantly greater for older adults. Several subsequent studies have also replicated the finding that older adults gain more than younger adults from bimodal conditions compared to unimodal conditions (Peiffer et al., 2007; Mahoney et al., 2011, 2012). However, when the inputs are unrelated or conflicting, older adults are more negatively affected by the distracting information than young adults (Setti et al., 2011). For example, Poliakoff et al. (2006) tested a crossmodal cuing task that required young and older participants to report the spatial location of vibrotactile stimuli while visual stimuli were presented in either congruent or incongruent locations. Older adults were significantly more distracted by spatially incongruent visual stimuli compared to young participants.

Thus, while older adults can gain from MSI-based facilitation effects, they also exhibit stronger debilitation under MSI-based distraction conditions. What explains these divergent effects? There are likely several contributing factors. First, with deficits in unimodal perception, older adults are likely compensating by relying on the additional information provided by MSI. Consider Mahoney et al. (2014), who found that around 75% of older adults tested evidenced MSI enhancement (faster performance in visual-somatosensory testing conditions than in either visual or somatosensory alone conditions). However, the remaining 25% did not show MSI enhancement; rather, their MSI responses were as fast as their unimodal somatosensory responses, which were in turn similar to speeds reported for younger adults in prior literature (e.g., Laurienti et al., 2006; Mahoney et al., 2011). This faster group of elders did not need to draw on multisensory benefit given their command of unimodal processing, whereas the slower older adults drew on the second sensory modality to reinforce the original weaker one.

Second, when vision is one of the modalities being integrated, older adults often appear to prioritize it over audition and touch. Throughout this paper, we will be using the phrase visual dominance to describe this over-reliance on visual processing over other sensory modalities. It is important to point out that visual processing factors dominate over the other sensory modalities for all normally sighted humans, not just older adults (Posner et al., 1976). The findings of this literature review is that visual dominance holds greater sway for older adults when compared to younger adults. Although this is not readily apparent when information from the two modalities provide synchronous information, this bias emerges more clearly during times of conflict (i.e., increased distraction for older adults when visual information conflicts with tactile). This age-related increase in visual capture has been well-explored in crossmodal attention research, indicating that older adult processing is dominated by the visual components under MSI testing conditions (Thompson and Malloy, 2004; Mahoney et al., 2012). A key study in this regard is Diaconescu et al. (2013), who assessed age group differences in brain activity during crossmodal processing using magnetoencephalography (MEG). In the task, younger and older adults made categorization judgments for stimuli presented either unimodally (auditory or visual) or bimodally (auditory + visual). Although both age groups benefited from the addition of visual inputs compared to auditory alone, the benefit was greater for older adults. MEG recordings indicated a probable explanation: multisensory gains in the older adults were associated with increased medial prefrontal and posterior parietal activity, with the latter region implicated in visuo-motor performance. The authors characterized these results as indicative of visual dominance, a compensatory mechanism in which older adults draw more heavily on visual processing brain regions to offset age-related reductions in cortical and subcortical gray matter volume.

Prioritizing visual over somatosensory information may help explain why older adults exhibit balance control issues and increased falls (Kannus et al., 2005). Standing balance is compromised in older adults (Laughton et al., 2003), a finding which has been attributed to their overemphasis of visual processing factors (Horak et al., 1989). When attempting to maintain an upright posture, older adults exhibit increased susceptibility to sway based on visual manipulations (Wade et al., 1995; Prioli et al., 2005; Toledo and Barela, 2014). It is not surprising, then, that walking is also compromised in aging. Efficient walking requires a complex integration of visual, tactile, and vestibular inputs with motor sequencing commands. Yet when walking, older adults tend to focus more on the ground and thus draw more heavily on optic flow information (Anderson et al., 1998), with greater attention to visual factors (Sparrow et al., 2002) which may reflect the decreased processing power of proprioceptive systems (Huitema et al., 2005). Fall-prone older adults appear to rely heavily on visual processing due to interoceptive processing failures (Barrett et al., 2013), and MSI control failures in older adults have been linked to both poor balance (Stapleton et al., 2014) and increased falls (Mahoney et al., 2014).

Yet this visual dominance affecting older adults is neither inevitable nor fixed. Recent research has shown that MSI in older adults is highly plastic, and MSI-based training can result in improved walking and balance performance (Setti et al., 2014). For instance, Merriman et al. (2015) explored whether a balance intervention program could help MSI problems in older adults. They found that older adults benefited from the intervention, with improved balance control when compared to similarly matched peers. Importantly, fall-prone older adults showed significant improvement in MSI performance based upon the balance control intervention. Thus, behavioral intervention holds the promise of offsetting the visual dominance exhibited by older adults and reducing the potential for falls.

### The Body Schema and Peripersonal Space

The body schema and peripersonal space are concepts used to explain how humans understand the physical body and the external space immediately surrounding it. While these terms are treated as functionally distinct, they share many similarities and in fact are often used somewhat interchangeably. The body schema is the internalized ‘map’ of spatial relations for one’s own body based on kinesthetic, proprioceptive, and tactile information, which is ultimately used to facilitate successful action; peripersonal space, on the other hand, is the space immediately surrounding the body through which we interact with the surrounding environment. Ambiguity in the exact boundaries of these constructs is understandable: when examining the understanding of physical actions (a body schema question), that movement necessarily takes place in peripersonal space, and the influence of that space is difficult to disentangle from the body schema itself (c.f., Cardinali et al., 2009 for a full review).

Both the body schema and peripersonal space are essential to embodiment theory, for they form the foundation for the intersection of sensory experience and bodily action (Borghi and Cimatti, 2010). Both the body schema and the formation of peripersonal space are mediated through MSI, the result of combining weighted signals from visual, tactile, and proprioceptive domains (Blanke, 2012). Additionally, both allow for multiple action-perception relationships, for the capacity to perform bodily actions requires an awareness of one’s own body in space (Reed and Farah, 1995). Accordingly, the body schema and peripersonal space are plastic in nature, and can flexibly adapt to differing physical environments and bodily demands.

It is reasonable to expect that aging will affect the body schema and peripersonal space given that they are based on underlying factors that are vulnerable to aging. First, they represent the holistic integration of multisensory inputs within the body’s spatiality (Maravita et al., 2003), a process known to change during aging. Second, body schema mapping and the localization of objects in peripersonal space necessitate accurate proprioception (Walsh et al., 2011; Proske and Gandevia, 2012), which is known to decline with advanced age (Goble et al., 2009). Finally, the body schema is continuously updated by vestibular signals (Lopez et al., 2012), and vestibular control declines with advanced age (Alexander, 1994). Taken together, one would expect profound changes to the body schema and peripersonal space with advanced aging.

Studies examining peripersonal space in young adults have sought to identify perceptual differences between near-body and far-body (extrapersonal) space. One common difference is that in peripersonal space, young adults deploy attention asymmetrically, over-emphasizing the left visual field over the right. This is thought to occur because spatial attention in peripersonal (but not extrapersonal) space is primarily controlled by the right-hemisphere (Fink et al., 2001; Bjoertomt et al., 2002). On tasks such as line bisection, in which participants indicate the center point on a straight horizontal line, it manifests as a slight leftward bias when the line is presented in peripersonal space (Jewell and McCourt, 2000). In extrapersonal space, where the asymmetry does not occur, young adults bisect lines at veridical center or with a slight rightward bias (Varnava et al., 2002; Garza et al., 2008). Older adults, on the other hand, exhibit a rightward bias in peripersonal space (Fujii et al., 1995; Jewell and McCourt, 2000). This occurs even when manual abilities are eliminated and when implicit tasks are used to account for the possibility of strategy differences (Barrett and Craver-Lemley, 2008). This may be occurring because older adults do not have right-hemisphere dominance for coding peripersonal space, requiring activation of the left hemisphere to compensate (Cherry et al., 2005; Chen et al., 2011).

Ghafouri and Lestienne (2000) used a sensorimotor experimental design to evaluate peripersonal space representations in older adults, having participants draw imaginary ellipses in the air in different planes in the space in front of their bodies. The authors argued that representations of 3D space can be revealed by examining goal-directed movements within that space: if space is not represented veridically, the movements should reflect those inaccuracies. The authors compared the abilities of young and older adults to orient ellipses in three different planes by first showing participants a template and asking them to reproduce it. In comparing the plane of motion of each participant’s finger, they found that older adults had smaller plane volumes than young adults, indicating a compression in the representation of peripersonal space. Furthermore, by comparing performance across the different planes, they were able to show that the motor component was not responsible for the errors; rather, older adults were representing the spatial coordinates incorrectly.

Although these behavioral tests are in line with peripersonal representations changing with age, not all studies find age differences. Older adults have been found to exhibit leftward biases when drawing or arranging items in peripersonal space, similar to young adults (Barrett and Craver-Lemley, 2008). While these measures were implicit tasks (unlike line bisection), other studies using seemingly implicit dependent measures such as ellipse drawing have shown these differences, suggesting that changes in performance with age are not just due to changing strategies. It is unclear what is causing these divergent results, and further research is needed to clarify why peripersonal space representations sometimes, but not always, show age-related changes.

With regard to the body schema proper, little research has directly explored how it may be affected by aging. Gilpin et al. (2015) recently tested how osteoarthritis affects the body schema of older adults. In their study, arthritis sufferers and non-arthritic controls viewed live video feed of their own hands while their hands were manipulated to be ‘stretched’ (the imaged hand was digitally elongated while physically pulled), ‘shrunk’ (digitally contracted while being pushed), or presented without manipulation. Participants were then asked to resize digital photographs of their hands to veridical sizes. Analyses indicated two key findings. First, the non-arthritic controls exhibited the expected shrinking and stretching illusion, whereas this manipulation was muted in the arthritis sufferers. Second, arthritic seniors reported smaller hand sizes than non-arthritic controls, suggesting that pain contracted the spatial representation of the hand. Although this study indicates the alteration of the body schema under a common older adult disability, the results point to body schema plasticity based upon age-related pathology (i.e., arthritis) rather than aging itself. However, it indicates that changes in the body and physical ability have the capacity to alter the body schema. Further research is needed to determine how the body and physical changes that accompany healthy aging also alter the body schema.

## Embodiment in Mental Representations

Having detailed the age-related changes to sensory-level processing, we turn now to how aging alters mental representations based on that sensory processing. The embodied cognition framework regards mental concepts as either heavily interactive with sensorimotor systems or as directly related to sensorimotor function (Glenberg, 2015), whereby concepts are instantiated by reactivations of sensory- and/or action-based neural pathways (Barsalou, 1999, 2008). As such, mental representations are emblematic of sensorimotor function. This is in contrast with the traditional cognitive science model, wherein perceptual processing of sensory inputs occurs through mental representations that are understood as abstract and amodal symbols for the represented percepts (Fodor et al., 1975; Pylyshyn, 1984). To explore how aging affects the influence of embodiment on mental representations, we will examine age-related differences in mental imagery, motor imagery, and action observation.

### Mental Imagery

Mental imagery is the creation, maintenance, and activation of an internal representation. Although seemingly simple in definition, mental imagery depends upon multiple cognitive subcomponents including sensory processing (the image is typically either visual or auditory in nature), cognitive processing (the image is understood in terms of its physical construction and/or semantic content), and working memory (mental images can be reproduced, maintained, and elaborated upon; Kosslyn, 1995). Mental imagery shares a similar underlying neuroarchitecture with perceptual processing, such that imagining an object elicits similar brain activations as directly perceiving the object (Kosslyn et al., 2001; Wilson-Mendenhall et al., 2013). This close relationship between the perceptual processing and mental imagery suggests that older adult deficits in unisensory and multisensory processing may yield similar deficits in mental representations.

Perhaps the canonical experimental paradigm for mental representation study has been the mental rotation task, first developed by Shepard and Metzler (1971). In such tasks, participants are shown an object on a screen and are required to mentally rotate it, typically responding by selecting the target angle from an array of variously rotated items. Early research on age-related differences in mental rotation found that older adults committed greater errors and yielded slower performance in mental rotation compared to young adults (Cerella et al., 1981; Hertzog and Rypma, 1991), indicative of a generalized decline in mental imagery (Craik and Dirkx, 1992). However, later work found that age effects on mental rotation are not a global failure, but rather are evident primarily under the cognitive strain of difficult rotation angles and increased complexity (McDowd and Craik, 1988; Inui, 1997; Sit and Fisk, 1999). Indeed, recent work from an embodied cognition perspective has found that both older and younger adults have mental representations that are similarly grounded in sensory processing (Vallet et al., 2011, 2013). Age-related deficits in mental rotation, when present, may therefore reflect the increased cognitive demands of spatial processing.

The mental rotation of the body is of particular importance to embodied cognition theory given that it represents the mental manipulation of the internalized body schema (Kaltner et al., 2014). Research with young adults has indicated that such imagery is constrained by body spatiality and bodily actions (Amorim et al., 2006; Steggemann et al., 2011). Importantly, older adult performance in mental rotation worsens when the target shape is a body. Devlin and Wilson (2010) assessed the effect of aging on mental rotations with three different imagery types: an alphanumeric stimuli set (5 or *F*), a 2D image of a single hand, and 3D whole-body image. Although age effects were evident across all three image types, older adults were significantly worse performing the whole-body rotations, with increasing reaction times and errors when rotating at larger angles. In this case, the whole-body stimuli represented not only a more complex stimuli set, but one intimately linked with the body schema.

While it may not always be clear whether older adult difficulties in whole-body mental rotation are due to task complexity or poor body representations, there is evidence that egocentric perspectives are specifically problematic. First, when the mental rotation requires an egocentric perspective (such as rotating a body image by means of one’s own body), older adults show deficits compared to young adults (Jansen and Kaltner, 2014; Kaltner and Jansen, 2016). Second, studies in spatial learning have found that older adults show marked deficits in learning spatial environments when operating through an embodied and first-person perspective (Inagaki et al., 2002; Borella et al., 2015). For instance, Yamamoto and DeGirolamo (2012) had young and older participants encode landmarks in a simulated environment either through a first-person or third-person perspective. When participants were later asked to reconstruct the spatial layouts, older adults were less accurate than young adults in the first-person, but not the third-person, encoding conditions. Finally, when older adults are required to make mental rotations of hand stimuli, their performance is significantly worse compared to younger adults when the rotations required an egocentric (as opposed to allocentric) rotation strategy (De Simone et al., 2013). Taken together, we can interpret these multiple instances of age-related decreases in first-person perspective to indicate that older adults may be downgrading their bodily inputs, resulting in decreases in egocentric (first-person) processing and consequent increases in allocentric (third-person) processing.

### Motor Imagery

A subtype of mental imagery is motor imagery, the mental simulation of an action when there are no overt physical actions being made. Motor imagery is arguably a more complex variant of mental imagery for it includes an implicit action plan (Kosslyn, 1987). Motor imagery is integral to embodied cognition because it combines external representations of the environment and peripersonal space with representations of the body schema to construct simulations. Motor imagery is critical to efficient intentional actions, because it serves as an internal simulation of actions that can moderate overt motoric action (Wolpert, 1997; Wolpert and Kawato, 1998) by working as forward-models in the planning of physical actions (Beauchet et al., 2010).

Our understanding of motor imagery has been greatly influenced by Jeannerod, who theorized that covert (mentally simulated) actions are neurologically similar to overt motor actions, such that both elicit comparable cortical activation patterns (Jeannerod, 1994, 2001). Indeed, there has been strong empirical support for this neurological overlap (Decety, 1996; Szameitat et al., 2007; Kraeutner et al., 2014). Motor regions activate when merely viewing action-based objects (Chao and Martin, 2000), and motor imagery elicits sensorimotor brain responses (Hauk et al., 2004). Jeannerod’s motor simulation theory is broadly compatible with the embodied cognition framework, and is conceptually akin to action-perception theories that similarly emphasize the close connection between sensation, action, and cognition (Hommel et al., 2001; Proffitt and Linkenauger, 2013).

Research indicates that motor imagery declines are evident in healthy aging (Personnier et al., 2008; Skoura et al., 2008; Saimpont et al., 2009). For instance, Gabbard et al. (2011) asked young and older adults to declare whether a target was within their arm’s reach after mentally simulating the action. Older adults overestimated their reach compared to young adults, which may reflect age-related deficits in brain regions critical to motor imagery (Munzert et al., 2009). Declines in motor imagery appear linked to handedness, with older adults exhibiting greater errors when operating with the non-dominant compared to the dominant arm (Skoura et al., 2008; Saimpont et al., 2009).

Perhaps not surprisingly, there are many similarities between the age-related declines in both mental imagery and motor imagery. Like mental imagery, the aging effect on motor imagery is also affected by both the complexity and perspective of the imagery. Under simple motor imagery conditions, older adults perform equivalent to young adults (cf., Saimpont et al., 2013). However, as the motor sequences to be imagined become more challenging, older adult imagery becomes less accurate compared to young adults (Skoura et al., 2005).

One specific form of additional complexity for older adults are first-person motor imagery visualizations. Mulder et al. (2007) had young and older participants report visualization strengths of their own or other’s actions. When older adults visualized their own first-person actions (as opposed to a third-person perspective visualization), the vividness in the images decreased. Importantly, this decline in the first-person perspective reflected the declining physical powers of the older adults: imagery vividness scores were correlated with measures of motor capacity and mental and physical speed measures. The effect of aging on motor imagery, therefore, is subtle: relatively preserved in cases of simple motor images without dynamic physical constraints, but degraded under more complex imagined action conditions (Personnier et al., 2008; Saimpont et al., 2013), particularly those involving egocentric perspectives. This result is consistent with theories of aging in which older adults suffer from a diminution of mental resources, resulting in increased difficulties from distraction under complex task requirements (Lustig et al., 2007).

Visual dominance of motor imagery in older adults has also been identified in neuroimaging studies. When using motor imagery, both young and older adults engage a network of neural pathways that include visual imagery and motor pathways as well as frontoparietal, subcortical, and cerebellar areas. However, activation intensity and specificity does differ by age group, suggesting that age effects in motor imagery reflect the changes to these brain networks (Saimpont et al., 2013). During motor imagery, older adult brains respond with larger and more diffuse activation patterns (Léonard and Tremblay, 2007; Nedelko et al., 2010; Sharma and Baron, 2014; Reuter et al., 2015), suggesting degraded or less-specific motor representations leading to compensatory brain recruitment (Zapparoli et al., 2016). When performing overt motor actions, older adults exhibit increased frontal and prefrontal (pre-SMA) activations during more cognitively complex actions, whereas when mentally simulating actions older adults exhibited increased activations in occipito-temporo-parietal areas (Zapparoli et al., 2013). This latter finding suggests that older adults rely more heavily on visual processing while performing motor imagery, consistent with our earlier finding of older adult over-reliance on visual processing in multisensory perception. Thus, behavioral evidence of visual dominance in older adults is matched by neuroimaging results suggesting increased activity in visual processing regions.

### Action Observation

Jeannerod’s (2001) motor simulation theory stipulates not only that motor imagery and physical actions share a common neural substrate – it also predicts that there is a similarly shared activation network when observing such actions in others. Indeed, there is mounting evidence indicating that merely observing someone performing an action results in the activation of the sensorimotor pathways responsible for producing that action (Buccino et al., 2004; Lui et al., 2008). This shared cortical activation pattern may offer a twofold advantage: allowing superior anticipation of the actions of others and providing information on one’s own actions (Jeannerod, 2001; Jeannerod and Anquetil, 2008).

Given the age-related decline in mental and motor imagery, it is not surprising that older adults do not benefit from action observation to the same extent as young adults. Aging decreases the efficiency in learning and then executing novel motor sequences (Coats et al., 2013) with specific losses for action observation (Maryott and Sekuler, 2009). For instance, Maguinness et al. (2013) explored age-related differences in a task in which participants viewed videos of either a hand or a full body lifting weighted boxes and had to indicate the amount of weight being lifted. Older adults were overall less sensitive for weight prediction than young adults, but particularly so when there were fewer visual details on the biomechanics of the lift. Accordingly, the authors argued that older adult performance declines indicate that seniors relied on a more visual-based (rather than felt-based) strategy.

Older adults may rely on visual processing components because their internal action models are degraded (Boisgontier and Nougier, 2013). Diersch et al. (2012) explored this possibility with a task assessing differences between young and older adults in the temporal parameters of action prediction. In the task, participants were shown video clips of everyday action sequences with the scenes interrupted by a black-screen occluder. After the occluder was removed, participants rejoined the original scene, although the timing parameters were manipulated such that the pre- and post-occluder scenes were either temporally continuous or discontinuous with the preceding scene. Participants were tasked with identifying the amount of time that had been altered based on the occluder. Older adults were less accurate than young adults in predicting the time course of another’s action, a result that the authors argued indicated a specific age-related decline in action representations. More recently, the same research group found that older adults exhibited increased activity in visual regions when performing action prediction (Diersch et al., 2013), with more diffuse brain activity indicative of less-specific internal models of motor actions (Diersch et al., 2016).

## Embodiment in Perception and Action

Our review thus far has focused largely on the bottom-up factors (i.e., physiological systems and sensory processing) and the mental representations that underlie embodied cognition. We have found that aging affects these systems profoundly, with a common finding of visual prioritization over somatosensory processing in older adults. In cases of multisensory perception, older adult deficits in unisensory processing typically result in gains in multisensory inputs, although visual processing is dominant. A similar tendency toward visual dominance occurs in mental imagery (most especially in motor imagery and action-perception), for visual processing factors dominate the motoric representations in older adults. Thus, basic sensorimotor deficits in older adults result in increased reliance on visual processing over other sensory modalities, and this visual dominance alters the nature of action-based mental representations. We now turn to how bodily action alters perceptual experience, a topic that is central to the embodied cognition framework (Glenberg et al., 2013).

### Action-Specific Theory of Perception

A central tenet of embodied cognition is that the mind and the body have reciprocal influence: not only can the mind control and direct the body as traditionally thought, but the body can also direct and influence the mind through its movement, posture, and sensory input. Embodied cognition theorists have long argued that action alters perceptual judgment, and research has confirmed that perceptual judgments of object size and distance correlate with our capacity to act upon the object (Proffitt et al., 2003; Kirsch and Kunde, 2013; Linkenauger et al., 2013). This close link between perception and action is central to both embodied cognition theory (Proffitt, 2006; Glenberg et al., 2013) and ecological theories of perception (Richardson et al., 2008).

Witt (2011) has formalized this general dictum into a specific theory on how action affordances can alter spatial cognition. Her action-specific account of visual perception argues that visual information of an object (in terms of object size or distance) is scaled relative to an observer’s effort or ability to effectively interact with that object (Witt et al., 2004). Thus, perceptual appraisals are always scaled as a phenotypic expression of bodily capacity (Proffitt and Linkenauger, 2013) made in relation to action affordances (Kirsch and Kunde, 2013). In line with the action-specific perception theory, physical capacity of the perceiver has been found to be related to perceptual scaling, presumably to maximize efficiency in motor response. Several studies have found that athletes exhibit perceptual recalibrations that correlate with their performance when playing sports such as tennis (Witt and Sugovic, 2010), golf (Witt et al., 2008), softball (Witt and Proffitt, 2005), and football (Witt and Dorsch, 2009).

Given that the older adult body is limited in its physical capacity, it is reasonable to expect age-related changes in perceptual scaling. In an early study by Bhalla and Proffitt (1999)^2^ on the relationship between aging and perceptual capacity, younger and older participants provided slope estimates while looking up from the bottom of variously angled hills. Young adult slope estimates were steeper when placed in physically demanding conditions, a result consistent with the action-specific perception hypothesis. However, older adults provided steeper hill slope estimates than young adults even under typical physical conditions, suggesting that their reduced physiological potential led to perceptual bias. Perceptual scaling tasks have also found that older adults are sensitive to environmental impediments that would complicate walking (Sugovic and Witt, 2013), essentially indicative of increased cautiousness. Similar findings of visuo-motor cautiousness have been documented in gait analyses of older adults (Grabiner et al., 2001; Owings and Grabiner, 2004) and shoulder rotation movements when walking through door apertures (Hackney and Cinelli, 2011).

What explains this increasing cautiousness in the action-specific perception responses of older adults? While the answer is not yet clear, the action-specific account of visual perception would point to the physical limitations of older adults as determinative to perceptual scaling. If true, the number of potential physiological factors affecting the older adult perception-action relationship may be surprisingly broad. Action-specific perception research has generated a dizzying array of physical factors vulnerable to aging that can influence task performance, including strength (Linkenauger et al., 2009), pain levels (Witt et al., 2009), and even respiration capacity (Daviaux et al., 2015). All these factors tie directly to the changing bodies of older adults. Consider the more speculative possibility that the metabolism of older adults might affect their action-perception relationship. It is known that giving participants sugary drinks has been found to decrease judgments of hill steepness (Schnall et al., 2010), presumably due to altered energy levels (although cf., Durgin et al., 2012). Older adults have marked declines in energy intake levels (Vaughan et al., 1991) and metabolism rate decreases (Wilson and Morley, 2003). The cause for this age effect on the regulation of metabolism is diverse, ranging from the delayed absorption of macronutrients and to reduced sensitivity of taste and smell (Roberts and Rosenberg, 2006). This raises the intriguing possibility that such seemingly tangential physiological and/or sensory factors could alter the perceptual judgments of older adults.

### Tool Use

Embodied cognition argues not merely that the body and bodily action is formative toward cognition and perception, but that the mind itself is unconstrained by the physical boundary of the brain and is extended out into the body in action (Noë,, 2009; Clark, 2010). Accordingly, it is logical to assume that the body may not be the only tool for mind-extension. Indeed, embodied cognition theorists have argued that the mind extends itself through a wide array of tools; this goes beyond mere physical objects and includes even metaphorical tools such as cultural artifacts (Malafouris, 2004; Wilson, 2010) and language (Mirolli and Parisi, 2009; Borghi et al., 2013).

The study of how tool use is represented in the brain began largely with primate research using single-cell recordings of neuronal sensitivity. Such work has found that when monkeys use tools, the receptive fields of neurons normally coding the spatial extent of the hand became extended into the more distant space of the tool’s reach (cf., Baccarini and Maravita, 2013). An early report indicated that when monkeys were trained to retrieve distant food using a rake, the receptive fields for bimodal (visual and somatosensory) neurons within the postcentral gyrus elongated to include the functional reach of the rake (Iriki et al., 1996). Consequent work has indicated that such tool-based changes to neuronal function are evidence of the plasticity of body representations and the coding of spatial relations (Obayashi et al., 2001; Hihara et al., 2006). Collectively this research indicates that the body schema is instantiated in the brain through multimodal (typically visual and tactile) coding that is subject to alteration based upon tool use (Maravita and Iriki, 2004).

Studies with human participants using tools have found similar results of body schema adaption under both behavioral (Maravita et al., 2002) and neuroimaging (Järveläinen et al., 2004; Peeters et al., 2009) testing conditions. When using tools, peripersonal space becomes extended into extrapersonal space (Berti and Frassinetti, 2000; Farnè and Làdavas, 2000; Maravita et al., 2001; Farnè et al., 2007). Cognitive processes alter in accordance with tool use, as both visual attention (Farnè et al., 2005; Witt and Brockmole, 2012) and memory (Davoli et al., 2012a) become refocused to the space at the functional end of the tool. For instance, participants report the distance of targets to be closer to themselves when wielding a tool directed to the target (Reed et al., 2010). This spatial-contraction effect is evident even when participants merely intend to act upon the target with a tool (Witt et al., 2005) and when the tool offers only remote access (Davoli et al., 2012b).

How might aging affect the ability to incorporate a tool into the body schema? One source of evidence comes from pantomime studies, in which participants are asked to identify or act out a physical action (e.g., “show me how to cut with a knife”). In a manner consistent with our earlier review of action observation research, pantomiming research has found that older adults exhibit general declines in pantomiming human actions (Cavalcante and Caramelli, 2009), especially when the pantomiming involves use of a tool (Mozaz et al., 2002, 2009). When pantomiming tool use, older adults make a characteristic body-part-as-object (BPO) error, wherein body parts rather than simulated actions are used to represent tool use (Ska and Nespoulous, 1987; Peigneux and van der Linden, 1999). For example, when asked to pantomime scissors, older adults are more likely to represent the index and middle fingers as scissor blades than to grip imaginary scissors handles. These age-related increases in BPO errors may simply reflect the cognitive demands of mimicking a tool-wielded action (Cavalcante and Caramelli, 2009; Maryott and Sekuler, 2009; Mozaz et al., 2009), a pantomime complicated by required mimicry of both the motor intention and detailed hand kinematics. Conversely, older adult deficits in pantomiming may represent a specific deficit, akin to the ideomotor apraxia common to stroke patients (Mizelle and Wheaton, 2010). Yet direct empirical evidence has been minimal.

Costello et al. (2015) recently examined whether aging affects the spatial-contraction effect that occurs during tool use. In their first task, young and older adults were shown target circles at near-body distances (ranging from 44 to 89 cm away) and asked to estimate their distance after pointing to the target either with their hand or with a reach-extending tool. Only the young adults displayed the expected tool-based spatial-contraction effect, in which the targets appeared closer when the participants were using the tool. In a second task, distance estimates were derived for far distances (ranging from 3.4 to 25.3 m away) after either pointing with the hand or with a laser pointer, which has been found to produce spatial contraction (Davoli et al., 2012b). Similar to the first experiment, only the young adults displayed the expected spatial-contraction effect when using the laser pointer on the targets. Taken together, the two tasks indicate a generalized phenomenon in which older adults fail to exhibit tool-based plasticity of body representations.

What might explain older adults’ inability to incorporate tools into the body schema? If we assume that tool use is a complex function with multiple cognitive, sensory, and motor components, age effects at lower sensorimotor levels may disrupt the overt performance of using a tool. After all, tool use requires the smooth integration of visual and tactile inputs, and as already discussed, older adults struggle in MSI-based tasks. There is evidence to this effect. Heuer and colleagues have investigated age-related differences in multisensory processing during tool use with a visuo-motor rotation paradigm. In such tasks, participants look at a display screen while moving a mouse cursor to a target, but vision of the hand movements themselves are hidden under an occluder. On some trials, the cursor pathway is diverted from the actual pathway of the hand movement. After the participants complete their reach and return to the home position, the pathway directions of either the cursor or the hand are displayed. Participants are then asked to modify the displayed pathways to the correct location for the remembered target location. Perhaps not surprisingly, under such conditions older adults commit greater errors than young adults (Bock, 2005; Heuer and Hegele, 2008; Heuer et al., 2013). But more importantly, older adults were more heavily influenced by the visual information of the monitor cursor than the tactile information of their own hand movements (Rand and Heuer, 2013; Heuer and Hegele, 2014). Thus, the lack of spatial contraction based upon tool use (Costello et al., 2015) may be due to the aforementioned visual dominance in older adults: because their sensorimotor calibration is more heavily influenced by visual processing, the proprioceptive feedback from the hand using the tool is accordingly diminished.

Secondly, tool use is typically performed with the hand, and there is evidence that the cognitive system of older adults is desensitized to their hands. Young adults appear to automatically orient attention toward their hands both immediately prior to and during a reach (Tipper et al., 1992; Meegan and Tipper, 1998). Older adults, however, do not orient toward the reaching hand; instead, attention remains centered on the space around the trunk (Bloesch et al., 2013). Older adults, in other words, have an attentional reference frame that is less sensitive to their hands, a finding that is consonate with a similar result discussed earlier: when older adults perform mental rotations of hand-based stimuli their errors were greatest for egocentric (as opposed to allocentric) rotations, suggesting that the older adults struggle to extract sensorimotor information of their own hands to assist their mental rotation of the target hand (De Simone et al., 2013). If sensorimotor information about the hands is less available during aging, it would be less likely that representations are flexible enough to easily incorporate tools into the body schema.

One final factor that may also contribute to older adults’ ability to understand and use tools is the type of knowledge necessary for this complicated task. Effective tool use relies on both semantic knowledge, information connecting the purpose of a tool to a to-be-acted-upon object, and mechanical knowledge, information about the functional allowances of a tool-based on the properties of both the tool itself and the to-be-acted-upon object (Lesourd et al., 2016). Additionally, tool use depends on reasoning skills that allow for mechanical knowledge to be drawn upon given the tools available and the situational demands (Reynaud et al., 2016). While chronological age is predictive of declines in both types of tool-related knowledge, the relationship is considerably stronger for semantic than mechanical. Further, cognitive functioning mediates age-related declines in semantic, but not mechanical, tasks (Lesourd et al., 2016). Thus, tool use failures in older adults may be partially explained by an inability to access knowledge about a tool’s purpose or the type of task a specific tool is used to complete, rather than an inability to access the action affordances of the tool.

## Are Older Adults Less Embodied?

This review has explored how the framework of embodied cognition can be applied to the study of aging by detailing research falling under three subdomains pertinent to embodied cognition: sensory perception (with a focus on MSI), mental representation (with a focus on motor imagery), and the effect of action on perceptual judgments. Embodied cognition theory predicts that as the body and sensory systems decline, there will be corollary changes in perception and cognition. Older adulthood is a critical period of physical decline, and therefore embodied cognition is a valuable theoretical prism by which to view aging (Vallet, 2015). As we’ve described in the prior sections, the aging mind-body relationship is indeed seen consistently across a variety of paradigms measuring different aspects of cognition. The results indicate that aging has profound effect on embodiment, as summarized below.

### Embodiment Changes in Older Adults

While aging is associated with a host of physical changes that produce a variety of effects on embodiment, a consistent finding in the literature is that older adults are influenced largely by visual processing over tactile and kinesthetic processing. This visual dominance effect was evident across all three subdomains we covered. Older adults are more heavily influenced by visual processing components during MSI tasks (Diaconescu et al., 2013), which may explain the increase in falls for older adults (Mahoney et al., 2014; Stapleton et al., 2014). Age-related failures in postural control have been attributed to older adults prioritizing visual inputs over other sensory modalities (Wade et al., 1995; Prioli et al., 2005) due to decreased efficiency in control over body position (Toledo AND Barela, 2014).

Similar instances of visual dominance in older adults have been found when performing tasks involving action observation (Maguinness et al., 2013; Boisgontier et al., 2014), again across a number of domains. Older adults have increased activity in visual processing regions during action prediction (Diersch et al., 2016) and greater reliance on visual over somatosensory signals on visuo-motor tasks (Rand and Heuer, 2013; Rand et al., 2013; Heuer and Hegele, 2014). Similarly, sensorimotor learning in older adults is more strongly directed by visual inputs compared to young adults (Teixeira and Lima, 2009). This over-reliance on visual processing may explain why older adults exhibit declines in spatial cognition: MSI is down-weighting tactile/proprioceptive inputs relative to visual inputs. In essence, due to age-related declines in physical capacity, a compensatory mechanism arises that increases the influence of visual processing, resulting in sensorimotor calibrations that are scaled with either distorted tactile/kinematic inputs, or the tactile/kinematic inputs are reduced in their weighted contribution. Older adults, in relying more heavily on a visual signal and less on an internal, body-based signal, are effectively less embodied than young adults.

An important corollary phenomenon is the age-related change in perspective-taking: older adults struggle when visualizing from their own first-person compared to a third-person perspective, displaying an insensitivity for egocentric awareness. This was primarily evident in motor imagery studies which have found decreases in multiple areas: in first as opposed to third person perspectives (Mulder et al., 2007), in whole-body mental rotation with egocentric requirements (Devlin and Wilson, 2010), and when mentally rotating hand-based stimuli (De Simone et al., 2013). Thus, the older adults are less ‘inhabited’ within their body and less comfortable when forced to imagine their body in alternative viewpoints. How does this relate to the aforementioned visual dominance effect in older adults? One, first-person perspectives would encompass the body state of the older adult. If older adults have deficiencies in access to their body states, then the visualizations that depend on that perspective would consequently falter. Two, if older adults are less embodied and more objectivist in their perspective, their increased visual processing would force them ‘outside’ rather than staying in the body. In that sense, mentally seeing the world in the third-person would likely be clearer to older adults compare to an egocentric perspective.

In short, the body’s influence on cognition alters in older adults based on their reliance on visual signals over bodily control. Embodied representations rely on externally originating visual and tactile sensory signals as well as internally originating proprioceptive and vestibular sensory signals. These signals will be weighted differently depending on the task and the quality of the signal. For older adults the internal signals are often degraded – proprioception declines with advanced age (Goble et al., 2009), as does vestibular control (Alexander, 1994). Although vision declines with age as well, it can be corrected to normal more easily than these other senses. While it is the case that normally sighted humans, not just older adults, prioritize vision (Posner et al., 1976), when creating embodied representations the visual signal can be balanced with internal signals. If internal signals are less reliable or less available, as they are for older adults, it may be the case that the visual signal becomes most strongly weighted regardless of the task.

This scenario would predict that older adults would be most likely to prioritize visual information because it is the most readily available and often the most spatially and temporally precise. However, in cases where vision is not the most reliable modality, vision should follow perception, not lead it. While that is quite difficult to accomplish in healthy older adults, it is found in some clinical populations. For example, bilateral parietal lobe damage (Balint’s Syndrome) can impair the ability to visually localize and attend to stimuli while leaving these abilities relatively intact in the auditory modality. Phan et al. (2000) had both healthy participants and a patient with Balint’s Syndrome localize visual and auditory stimuli that were presented simultaneously. Healthy participants were captured by the visual stimuli, which is expected given the spatial superiority of vision over audition. However, the patient’s performance was captured by the auditory stimuli, which is consistent with the prediction that the modality that is most reliable and accurate will be the one whose signal is prioritized. Given the myriad unisensory and physical declines that accompany aging, it is reasonable for vision to be the most reliable; thus, it would make sense for vision to be the default modality.

Note that a similar reliance on visual processing components over somatosensory processing has been identified in older adults suffering from Parkinson’s disease (PD) in order to compensate for their physical declines. Imaging studies have found that older adults with PD not only perform worse on motor imagery tasks, but also show increased brain activity in extrastriate visual areas (Helmich et al., 2007). The physical limitations of the patients, in other words, is compensated with additional neural resources drawn from visual processing. At the behavioral level, this manifests in increasing dependence on a visual processing strategy (Poliakoff et al., 2010). Accordingly, motor imagery declines in PD are typically localized to the physical actions most debilitated by PD (Dominey et al., 1995; Helmich et al., 2007). Finally, Conson et al. (2014) found that PD patients had increased difficulty in whole-body mental rotations when the rotation type required a somatosensory ‘feel’ for the rotated body stimuli in contrast to a more visual grasp. This finding is similar in kind to healthy older adult deficits in first-person perspectives in body schema.

Interestingly, while older adults display visual dominance, infants, and children may display the exact opposite effect – a tendency to favor physical factors above visual factors. For instance, Frick et al. (2009) had 5-year-olds, 8-year-olds, 11-year-olds, and young adults (*M* age = 37 years) perform a jigsaw puzzle while working a hand crank. In some trials, the crank rotation matched the expected rotation of the puzzle piece, whereas in other trials the crank rotation was in the opposite direction of the puzzle rotation. When the crank and puzzle rotations matched, performance was improved in the 5- and 8-year-old children, but not the older children or young adults, indicating that motor factors were weighted more heavily early in development. This is consistent with other studies showing that the mental imagery of young children is more heavily influenced by motor processes over visual components (Frick et al., 2005; Funk et al., 2005). Infant research using looking-measure paradigms have similarly found that physical exploration is critical for infants to perceptually discriminate visual scenes (Frick and Möhring, 2013; Möhring and Frick, 2013), suggesting that mental imagery is linked with motor expression from early on. Although there is debate on the age of onset for the visuo-motor matching (cf., Krüger and Krist, 2009; Frick et al., 2013), the evidence points to an increased influence of motor factors on perceptual judgments for infants and children. Thus, from a lifespan perspective, the integration of visual perception and action systems is complex, favoring motor influences early in life, a more leveled integration in young adult period, and diminished motor and increased visual influence in older age.

The claim of visual dominance in older adults is based on the preponderance of evidence that we have reported in this review, although not surprisingly there are empirical results that run counter to this theme. Recall that we earlier reported an age-related increase in MSI-based errors during the Sound-Induced Flash Illusion. Recently, McGovern et al. (2014) found that the greatest age effects were found in the fusion illusion (in which two flashes paired with one beep is experienced as one flash) compared to the fission illusion (in which one flash paired with two beeps is reported as two flashes). If the action-perception system of older adults is more heavily driven by visual processing factors, one would have expected performance to be worse in the latter condition. Additionally, results from studies testing the action-specific theory of perception, in which older adults judge hills to be steeper than young adults (Bhalla and Proffitt, 1999) and distances to be greater when walking is difficult (Sugovic and Witt, 2013), appear to run counter to visually dominated perception. If older adults were up-weighting the visual signal, environmental factors that impede physical movements should have less of an impact on perception, not more.

It is difficult to know how to interpret these contrary findings. One possibility could be that differing results are due to the relative demands of different tasks. When judging hill steepness or target distance, explicit judgments are required that may lead to age-related strategy differences, a problem which has been documented in other domains (Geary et al., 1993). These types of perceptual judgments also occur on a much larger environmental scale than tasks assessing peripersonal space, motor imagery, or action execution. Perhaps in these circumstances older adults are able to up-weight internal sensory signals to inform perception. It is important to note here that we are not arguing that older adults do not have access to internal sensory signals. Rather, we are suggesting that the internal signal is discounted relative to vision due to vision’s reliability.

### An Embodied Cognition Model for Aging

Our review has indicated that older adults operate with a model of embodied cognition that is differently weighted than younger adults: emphasizing visual processing components and deemphasizing bodily (tactile, kinematic, proprioceptive) factors. Although it is beyond the purview of this review article, a logical continuation would be to formally develop (perhaps via computational modeling) an explicit model of how aging alters the manifold factors operative in sensorimotor-cognitive processing. Developing such a model will be a difficult task given the range of potential age-related causal factors. First, there are a host of ‘non-embodied’ (as traditionally viewed) cognitive and brain factors that must play a role in how embodiment is altered in aging. As briefly stated in the beginning of this review, older adults exhibit a range of cognitive and brain-based changes. This review has focused almost exclusively on three subdomains pertinent to embodied cognition, but these subdomains are not independent of such factors. Second, social factors are likely operative in the mind-body relationship in aging, although almost no research has been conducted to determine this possibility. This is particularly important considering how negative societal views on aging (i.e., ageism) might alter performance of the older adults. If an older adult believes that he or she is out of shape or cognitively slow, we can expect that participant performance recorded through most behavioral measures will be affected (e.g., Levy, 2003). Third, older adult differences in embodied cognition may reflect cohort differences in strategy. Older adults perform tasks more conservatively, requiring increased evidence prior to making decisions (Ratcliff et al., 2004). It should be noted, however, that these age-related differences in response criterion may themselves simply reflect the age effect in embodied cognition.

We need to be cautious, then, with the concept of an age-related visual dominance. We do not want to say that in *all* tasks older adults will favor visual processing. Compensation can take multiple forms depending on the context, and in cases in which the visual inputs are minimized, we would expect that older adults will take advantage of motor factors. Consider Roski et al. (2014), who examined brain activation responses in young and older adults with two task conditions: a motor-based and a visual attention task. In the motor-based task, older adults had decreased activation for motor regions but greater in visual processing regions – an expected result given the visual dominance effect in older adults. However, in the visual attention task there was the opposite effect – activation increased for motor and decreased for visual processing brain regions. In short, older adults were displaying a dedifferentiated brain response in which the distinct functionality of these regions was less defined for older adults, perhaps due to compensatory recruitment. An age-related visual dominance effect, in other words, may simply be a compensatory draw on any and all available sensory inputs, with visual inputs being the quickest and most salient in most task conditions.

We can best understand this age-related change in sensorimotor control as a change to the internal model of older adults. The internal model is the mental calibration of many inputs, resulting in a controlled and purposeful response (Wolpert et al., 1995). Because older adults are vulnerable to cognitive, perceptual, physiological, and action-based deficits, the calibration of the internal action model is altered, with weaker physiological inputs and consequent over-reliance on visual processing. Several research groups have argued that aging results in degraded internal action models (cf., Cressman et al., 2010; Boisgontier and Nougier, 2013; Lafargue et al., 2013). Degradation in the internal action model may also explain the characteristic dedifferentiated brain response in older adults. If embodied cognitive effects in young adults reflect the efficient integration of sensory, cognitive, and motor systems, the older adult brain is characterized by the failure to recruit specialized neuronal modules and the adoption of a more diffuse cortical response (Mott et al., 2014; Roski et al., 2014). Research indicates that the older adult motor system is affected by dedifferentiation (Bernard and Seidler, 2012; Koppelmans et al., 2015; Reuter et al., 2015). Further, motor performance in older adults is affected by cerebellar changes, leading to ‘degraded internal models’ (Bernard and Seidler, 2014). Dedifferentiation, which is associated with a lack of specialized neuronal modules, may compromise the ability to calibrate internal and external sensory signals, resulting in inaccurate weighting. Inaccurate or altered weighting may explain the age-related behavioral changes in embodiment we’ve described in the review above.

### Future Directions

As we have highlighted throughout the review, virtually every area of aging would benefit from further research using an embodied cognition perspective. Given that, here we will highlight just a few areas of practical importance. One question that has not yet been explored is the age at which changes in embodiment begin to take place. If age-related changes are based on neural dedifferentiation or compensation, then it would be expected that the changes are gradual and positively correlated with age. Relatedly, given that there are individual differences in the onset and rate of neural change with age, there should be corollary individual differences on tasks that tap into embodied effects. Indeed, there is preliminary support for this, with older adults exhibiting wide variability in the relative impact of the body during visuo-motor performance (Bloesch et al., 2013). Not surprisingly, young adults also show individual differences, which are often overlooked by only examining group differences. By expanding age groups to include middle-aged individuals and testing for individual differences, we can begin to achieve a more nuanced understanding of how and when changes in embodiment occur across the lifespan.

It is clear that older adults have altered MSI, and that this impacts not just sensory processing but also higher-level cognition and physical ability. For example, there is a link between MSI and balance, with training on one factor improving performance on the other (Setti et al., 2014; Merriman et al., 2015). Given that falls are a concern for older adults, anything that can reliably improve balance and decrease fall risk is potentially meaningful. Unfortunately, there is still a paucity of research on this topic. The MSI conditions that have been trained are visual-auditory, but given the link between visual-tactile integration and action it may be the case that other types of MSI training would improve balance, and potentially other types of action as well. Visual-tactile integration is also important for successful tool use, which older adults struggle with compared to young adults. While many older adults experience benefits from the use of canes and walkers to assist in mobility, many others report that these tools hinder them and, in fact, increase their risk of falls (Bateni and Maki, 2005). If this struggle is caused in part by a decreased ability to integrate visual-tactile information about the mobility device and bring it into the body schema, training this type of MSI in older adults may be beneficial.

Embodied cognition claims that there is a causal link between physical ability and cognition, so it should not be surprising that studies have found correlations between older adults’ physical capacities and their perceptions (e.g., Sugovic and Witt, 2013). However, many of the studies explicitly examining the relationship between physical declines and the specific cognitive factors assumed to be impacted based on the predictions of EC (including the majority of the studies included in this review) are correlational, leaving unanswered the direction of the relationship or the potential for a third variable. An avenue in which this relationship has been explored experimentally is in fitness training with older adults, which has found that physical interventions such as aerobic exercise have the potential to improve cognitive performance across a variety of tasks, notably processing speed, and visuospatial ability (for recent reviews, see Bherer et al., 2013; Bamidis et al., 2014). Because these are variables that have been implicated in older adult declines on embodied tasks, improving cardiovascular health would perhaps improve embodiment in older adults as well. If this is true, older adults in better physical health should also show greater embodiment than older adults in poor health, a question which can begin to be answered by examining individual differences.

There are many avenues still available for exploration in applying the embodied cognition theoretical framework to understand aging. Indeed, it would appear that we are just scratching the surface of applications to aging, and already we are gaining a richer understanding of age-related declines and the causes of them. The history of gerontological research has focused on isolating and then assessing sensory, cognitive, and physical abilities; it is now more clearly understood that these three systems interact, and that assessing changes means assessing those interactions as well.

## Author Contributions

MC compiled original literature review and wrote primary manuscript. EB contributed to both literature review and manuscript preparation.

## Conflict of Interest Statement

The authors declare that the research was conducted in the absence of any commercial or financial relationships that could be construed as a potential conflict of interest.

## Footnotes

1 At its extreme theoretical variants, embodied cognition theory is both non-computational and non-representational (cf., Chemero, 2009), although this is not a universal position within the embodied cognition field (Clark, 1997). Given that we will be drawing upon a broad array of research in this review article, we employ a more inclusive variant of embodied cognition in which traditional cognitivist terms such as ‘mental representations’ are utilized.

2 Note that this work has been criticized as reflecting participant effects in how participants respond rather than actual measures of perceptual alteration (Firestone, 2013; see Witt et al. (2016) for a reply). Recently, Philbeck and Witt (2015) have offered a more measured action-perception model.
